# Cure Efficiency and Biocompatibility of an Iron-Based Coordination Complex as a Photoinitiator for Dental 3D-Printed Resins

**DOI:** 10.3390/jcs9010026

**Published:** 2025-01-08

**Authors:** Sharanya Singh, Mateus Garcia Rocha, Mario Alexandre Coelho Sinhoreti, Alexandre Carneiro Silvino, Dayane Oliveira

**Affiliations:** 1 Department of Restorative Dental Sciences, Center for Dental Biomaterials, College of Dentistry, University of Florida, 1390 Center Drive, Gainesville, FL 32610, USA; 2 Department of Restorative Dentistry, Dental Materials Division, Piracicaba Dental School, State University of Campinas-UNICAMP, 901 Limeira Ave, Areiao, Piracicaba 13414-903, SP, Brazil; 3 Professor Eloisa Mano Macromolecule Institute, Federal University of Rio de Janeiro, 2030 Horacio Macedo Ave, Cidade Universitária, Rio de Janeiro 21941-598, RJ, Brazil

**Keywords:** cytotoxicity, degree of conversion, dental photoinitiator

## Abstract

**Objective:**

The aim of this study was to evaluate the cure efficiency and biocompatibility of a novel iron-based coordination complex used as a photoinitiator in comparison to conventional ethyl (2,4,6-trimethylbenzoyl) phenylphosphinate (TPO-L) and camphorquinone (CQ) as photoinitiators in dental 3D-printed resins.

**Materials and Methods:**

Experimental dental resin formulations were prepared by blending 1:1 ratio of Bis-GMA and TEGDMA, to which 0.2 wt% of either the iron-based coordination complex or CQ were added, along with 0.2 wt% EDAB and 0.4 wt% IOD, and the TPO-L. The degree of conversion (DC) was measured using Fourier transform infrared spectroscopy (FTIR). Biocompatibility was assessed by evaluating the viability of L929 fibroblast-like cells using the MTT assay 24 h post-exposure. Statistical analyses included a two-way ANOVA followed by Tukey’s test for post hoc comparisons, with significance at *p* < 0.05.

**Results:**

The degree of conversion for the iron-based coordination complex (84.54% ± 1.69%) was significantly higher than that for the TPO-L (78.77% ± 1.25%) and CQ-based resins (73.21% ± 0.47%) (*p* < 0.001). The iron-based coordination complex and TPO-L resins exhibited significantly higher conversion than CQ-based resins (*p* < 0.001). Regarding biocompatibility, the cell viability test revealed that the iron-based coordination complex demonstrated the highest cell viability at 86.5% ± 10.24%, followed by TPO-L with 80.03% ± 11.07%. CQ showed the lowest cell viability of 51.29% ± 8.44% (*p* < 0.05). Tukey’s test confirmed significant differences between CQ and other photointiators (*p* < 0.05), while no significant difference was found between TPO-L and the iron-based coordination complex.

**Conclusions:**

This study introduces a novel iron-based coordination complex photoinitiator that demonstrates enhanced cure efficiency and comparable biocompatibility to TPO-L, while significantly reducing the cytotoxicity associated with CQ. Its longer absorption wavelength supports deeper layer curing, making it a promising alternative for dental 3D printing, particularly in bioactive scaffold applications requiring minimized cytotoxicity.

## 1. Introduction

Three-dimensional printing has become the latest technology to be utilized in dentistry, specifically the 3D printing of resin materials [1]. The use of dental 3D-printed resin includes provisionals, crowns, dentures, implants, orthodontic appliances, surgical guides, and diagnostic models [2]. Three-dimensional printing can also be utilized for bioactive scaffolds in periodontal tissue regeneration and bone regeneration [3,4]. These printed scaffolds have the potential of being used for patients who need tissue or organ transplants [5]. The 3D-printed scaffold imitates the extracellular matrix, favoring the growth of cells [4,5]. The field of dentistry uses two main technologies for 3D printing: stereolithography (SLA) and digital light processing (DLP) [2]. SLA and DLP methods utilize photopolymer resin (75% oligomers, 25% monomers, photopolymer initiator) to create dental 3D-printed resin materials in a layer-by-layer process [2]. When the photopolymer initiators are exposed to UV light in the 3D printing process, they create primary radicals that react with the oligomers and monomers [2]. In this process, the carbon–carbon double bonds belonging to the methacrylate moieties of the oligomer and monomer species are converted into carbon–carbon single bonds during the 3D process of polymerization [1]. The relative amount of double-bonds converted into the corresponding single carbon–carbon bonds is defined as the degree of conversion for the photopolymerized resin [1]. The SLA process does not result in a full degree of conversion during 3D printing, indicating the need for a post-curing process [1]. A lower degree of conversion results in lower physical properties that can have poor implications for delicate scaffolding biomaterials [6]. When the photopolymer considered as the oligomer–monomer mixture is incompletely polymerized, residual monomer or photoinitiator can be released to the media, resulting in potential cell DNA mutations or cell apoptosis when applied in bioprinting for scaffolds or surrounding tissues when used in the mouth [4,7].

The degree of conversion is highly influenced by the concentration and identity of the photoinitiator [1]. Currently, the most commonly used photoinitiator in dental 3D-printed resin materials is ethyl (2,4,6-trimethylbenzoyl) phenylphosphinate (TPO-L) [1]. TPO-L has an absorption wavelength band between 350 and 430 nm [1]. When exposed to light, TPO-L divides into two radicals, allowing for a faster polymerization reaction and a relatively high degree of conversion compared to camphorquinone [8]. Camphorquinone (CQ) is another photoinitiator used in 3D printing and is an alpha-diketone with a maximum absorbance of 468 nm [8]. CQ requires tertiary amines to act as co-photoinitiators that play the role of a reducing agent for the polymerization reaction [8]. CQ has a slower polymerization reaction when exposed to light as it only forms one radical and cannot polymerize as efficiently under UV light (200–400 nm), which is used in 3D printing [8]. Unreacted monomers and photoinitiators have been proven to have cytotoxic effects on live tissues [1]. TPO-L and CQ are not the most biocompatible photoinitiators to the cell when left unreacted by the curing process [1]. We then call into question a different photoinitiator with a higher degree of conversion and less cytotoxic effects on the cells to be used in the formulation of dental 3D-printed resin materials [1].

On the other hand, coordination complexes have been shown to provide many benefits over conventional photoinitiators. Firstly, coordination complexes have been shown to be more biocompatible with cells and less cytotoxic [9]. Radical polymerization reactions known as atom transfer radical polymerizations using coordination complexes result in a higher degree of conversion (with low amounts of unreacted monomers left after the polymerization), thus favoring lower cytotoxicity [9]. Therefore, the present study evaluated the cure efficiency and biocompatibility of a novel iron-based coordination complex used as a photoinitiator in comparison to the conventional TPO-L and CQ as photoinitiators in dental 3D-printed resin materials. The test hypotheses were as follows: 1-the use of a coordination-complex-based photoinitiator in 3D-printed resin-based materials would result in a higher degree of conversion (DC) compared to TPO-L and CQ, and 2- the use of a coordination-complex-based photoinitiator in 3D-printed resin-based materials would result in lower cytotoxicity compared to TPO-L and CQ. With promising results, we aim to demonstrate that using coordination-complex-based photoinitiators in 3D printing can improve the DC in dental 3D-printed resin materials, potentially reducing the need for post-curing processes and minimizing cytotoxicity. Inherently, this will provide a promising outlook for 3D-printed bioactive scaffolds regarding their strength and cytotoxicity to cells.

## 2. Materials and Methods

### 2.1 Iron-Based Coordination Complex Synthesis

**Figure 1.**
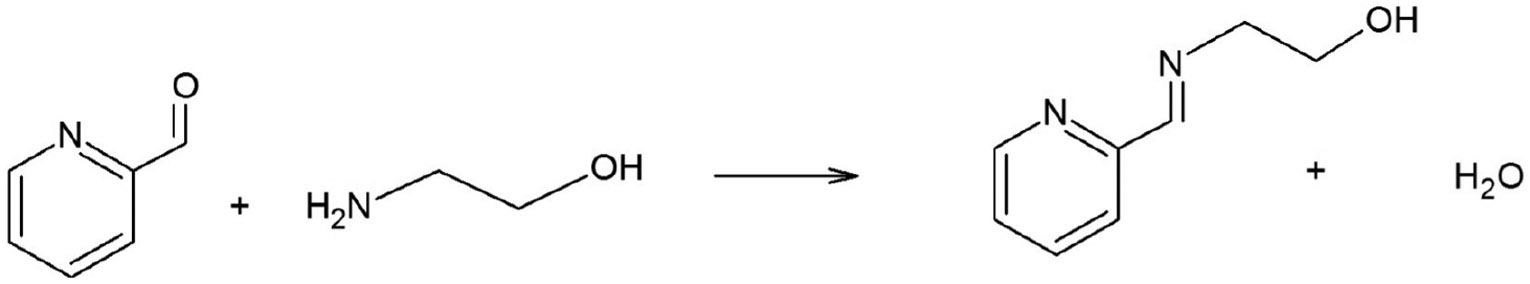
Reaction for synthesizing the imino-pyridine ligand.

**Figure 2.**
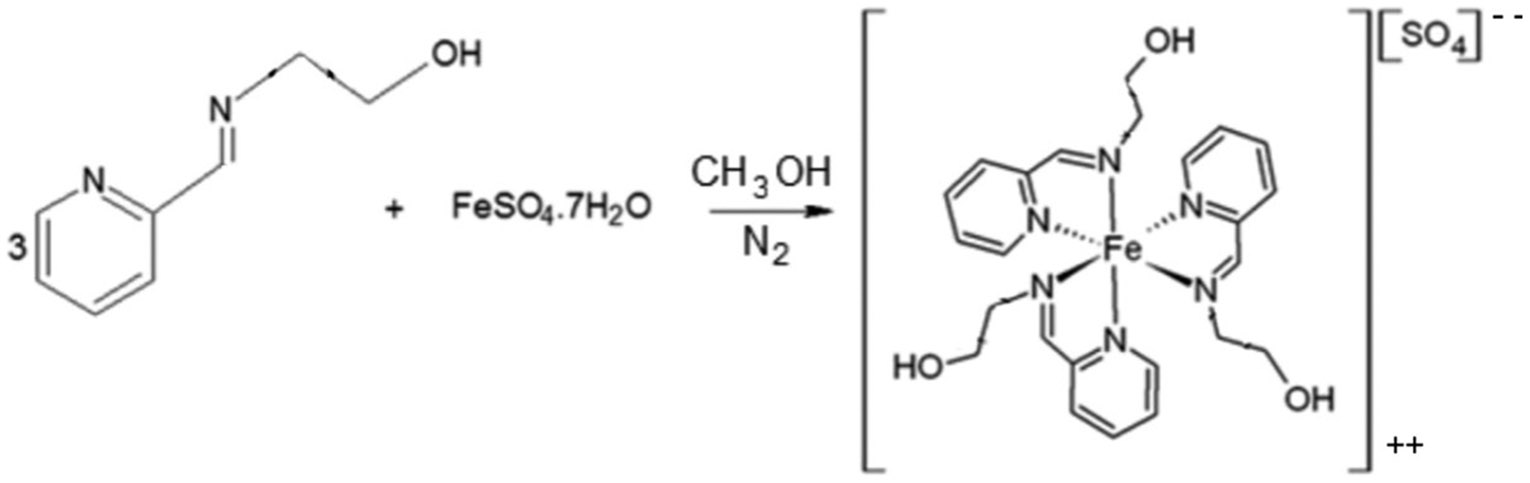
Reaction for synthesizing the Fe(II)-based coordination compound.

**Figure 3.**
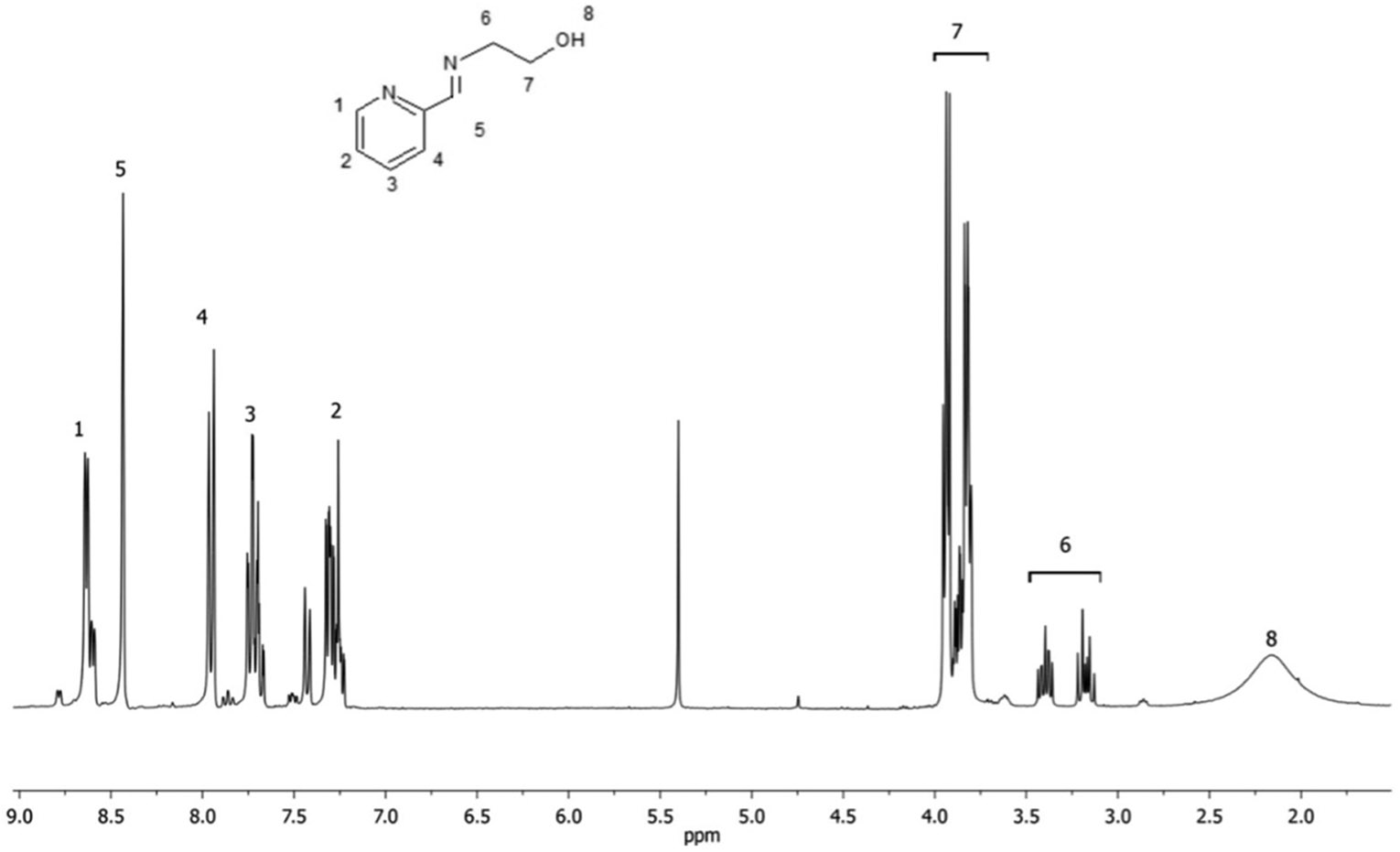
^1^H NMR spectrum for the imino-pyridine ligand identified as 2-[(2-pyridinylmethylene)amino]ethanol, as shown in Figure 1.

**Figure 4.**
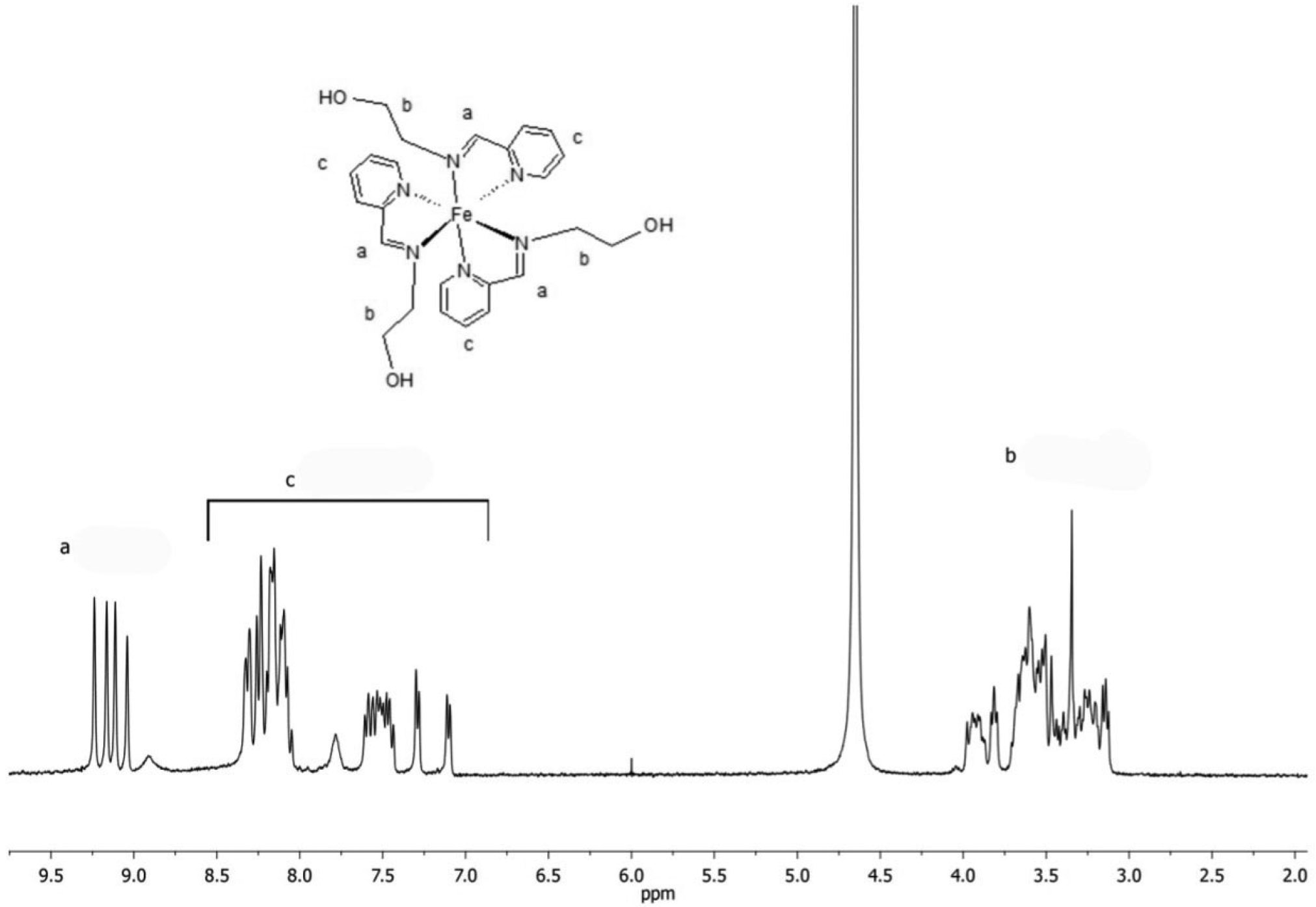
^1^H NMR spectrum for the newly Fe(II)-based complex identified as sulfate tris[2-[[(2-pyridinyl-kN)methylene]amino-kN]ethanol]iron(II), as shown in Figure 2, with the assignment of different types of protons: (a) imines; (b) aliphatics; and (c) aromatics.

**Figure 5.**
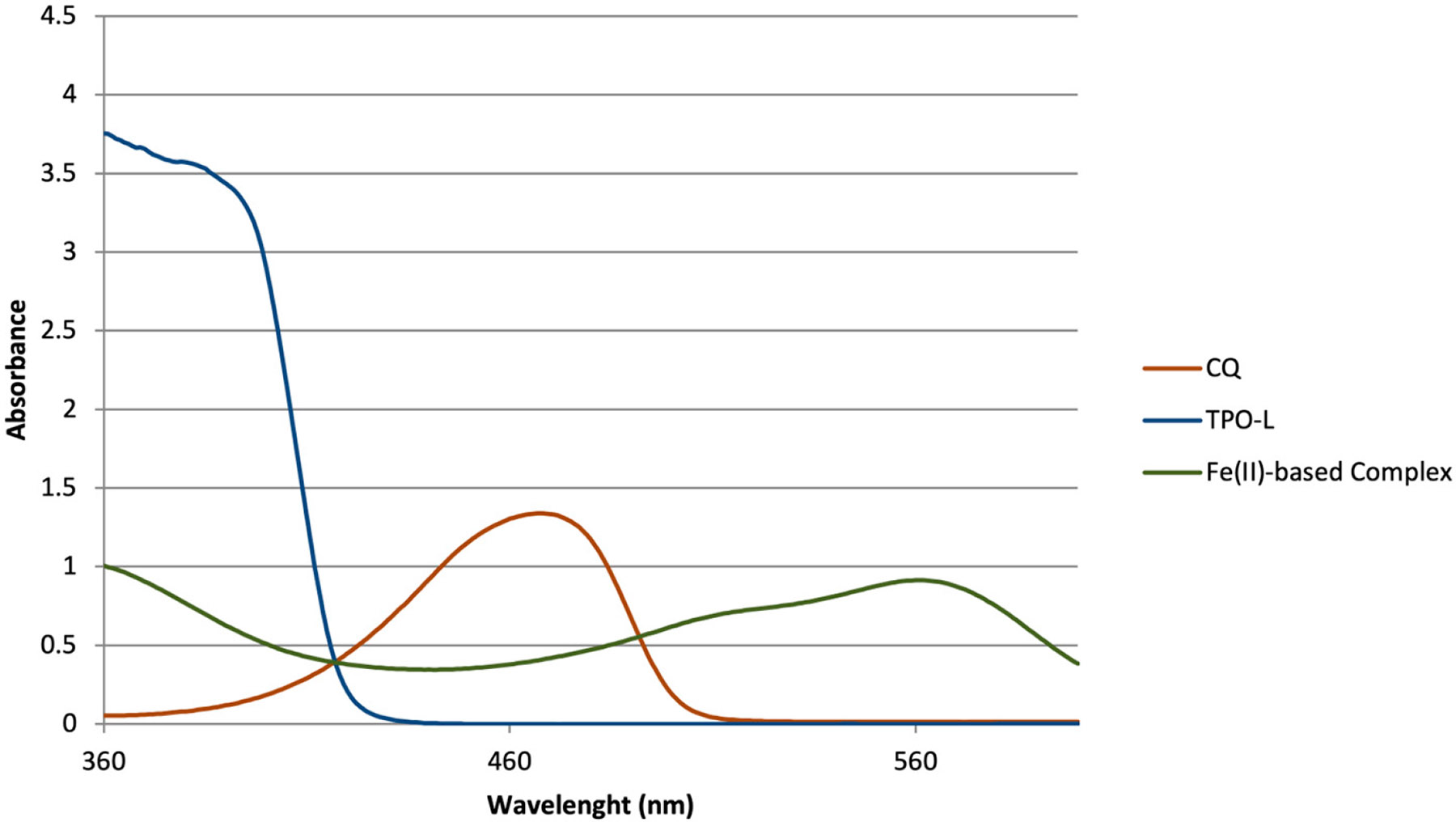
UV–VIS absorbance spectra for the newly Fe(II)-based complex compared to CQ and TPO-L.

Coordination compounds consist of a central metal atom or ion, called the coordination center, surrounded by molecules or ions known as ligands. The coordination compound tested in this study was an Fe(II)-based complex containing ligands derived from the iminopyridine unit. The ligands were prepared through the reaction of 2-pyridinecarboxaldehyde (Sigma-Aldrich, St. Louis, MO, USA) with 2-aminoethanol (Sigma-Aldrich, St. Louis, MO, USA), 1:1 molar ratio, in a temperature-controlled cold bath (Figure 1). Then, the synthesis of the Fe(II)-based complex was performed by reacting ferrous sulfate heptahydrate (Sigma-Aldrich, St. Louis, MO, USA) with the synthesized ligand, 1:2 molar ratio, under an inert nitrogen atmosphere (Figure 2). Methanol (Sigma-Aldrich, St. Louis, MO, USA) was used as the solvent in all reaction steps. At each step, characterization of each synthesized compound was carried out through nuclear magnetic resonance (NMR) spectroscopy (Varian Mercury VX 300 Hz, Agilent Technologies, Santa Clara, CA, USA)—using deuterated chloroform as solvent for the ligands and deuterated water for the iron complex—to confirm their chemical structures and molecular weights, as illustrated in Figures 3 and 4. The molecular structure of the coordination compound sulfate tris [2-[[(2-pyridinyl-kN)methylene]amino-kN]ethanol]iron(II) was defined as C_24_H_30_FeN_6_O_7_S with a molecular mass of 602.44 g/mol. Spectrophotometric analysis was also performed (U-2450, Hitachi High-Technologies, Chiyoda, Tokyo, Japan). For this analysis, each photoinitiator was diluted in 1 mL of >99.5% ethanol (Sigma-Aldrich, St. Louis, MO, USA) at the same concentration used in this study (0.2 wt.%). The spectra were collected using a quartz cell with a path length of 1 cm, as illustrated in Figure 5. The absorbance spectra of the Fe(II)-based complex exhibited absorbance across a broad range with a peak in the green region (500–600 nm), while CQ primarily absorbed in the blue region around 460 nm and TPO-L in the UV range.

### 2.2. Experimental Dental 3D-Printed Resin Formulation

Dental resin formulations were prepared by mixing a 1:1 ratio of bisphenol A-glycidyl methacrylate (Bis-GMA) (Sigma Aldrich, St. Louis, MO, USA) and triethylene glycol dimethacrylate (TEGDMA) (Sigma Aldrich, St. Louis, MO, USA) as the base matrix. Three photoinitiator systems were tested: (1) the novel iron-based coordination complex (COMFE); (2) TPO-L (Sigma Aldrich, St. Louis, MO, USA); or (3) CQ (Sigma Aldrich, St. Louis, MO, USA) as photoinitiators. All photoinitiators were used at a concentration of 0.2 wt% in the resin formulations. To CQ and COMFE, ethyl-4-dimethylaminobenzoate (EDAB, 0.2 wt%) (Sigma Aldrich, St. Louis, MO, USA) and diphenyliodonium hexafluorophosphate (IOD, 0.4 wt%) (Sigma Aldrich, St. Louis, MO, USA) were added as co-initiators. All steps described were mechanically mixed using a centrifugal mixing device (SpeedMixer, DAC 150.1 FVZ-K; Hauschild Engineering, Hamm, Germany) at 37 °C.

### 2.3. Degree of Conversion

The degree of conversion (DC) of the experimental dental 3D-printed resins was assessed using Fourier transform infrared spectroscopy (FTIR) coupled with an attenuated total reflectance (ATR) accessory (Nicolet iS20, Thermo Fisher Scientific, Waltham, MA, USA). Specimens of 5 × 1 mm (n = 5) were prepared using a Teflon mold. The resin blends were photo-activated through a polyester strip with a light-curing unit (Bluephase G4, at 1000 mW/cm^2^, Ivoclar Vivadent, Schaan, Liechtenstein) at a radiant exposure of 16 J/cm^2^, in immediate contact with the polyester strip. Following polymerization, the specimens were dry-stored for 24 h at 37 °C. The FTIR spectra of both the unpolymerized and polymerized specimens were recorded, with 16 scans performed at a resolution of 1 cm^−1^. The degree of conversion was calculated by comparing the ratio of absorbance of aliphatic C=C bonds (stretching vibrations at 1638 cm^−1^) to that of aromatic C=C bonds (stretching vibrations at 1608 cm^−1^) in both polymerized and unpolymerized conditions using the following formula:

(1) $$DC(\%)=\left(1-\frac{\left(\frac{Xa}{Ya}\right)}{\left(\frac{Xb}{Yb}\right)}\right)\times 100$$

where Xa (polymerized) and Xb (unpolymerized) represent the bands of the aliphatic double bonds, and Ya (polymerized) and Yb (unpolymerized) represent the bands of the aromatic double bonds.

### 2.4. Cell Viability Test

L929 fibroblast-like cells were cultured in Dulbecco’s modified Eagle’s medium (DMEM) (Gibco, Life Technologies, Waltham, MA, USA), supplemented with 10% fetal bovine serum (FBS) (Gibco, Life Technologies Waltham, MA, USA), 100 U/mL penicillin (Sigma Aldrich, St. Louis, MO, USA), and 100 mg/mL streptomycin (Gibco, Life Technologies). The cells were maintained in a humidified incubator at 37 °C with a 5% CO_2_ atmosphere. The cultures were monitored until they reached approximately 80% confluency, defined as the end of the logarithmic phase of growth.

To evaluate the cytotoxic effects of the tested materials on cell proliferation, sterile samples of the materials (similarly prepared as described in 2.3, n = 5) were incubated in the same media for 24 h to prepare conditioned media. L929 cells were seeded at a density of 3 × 10^4^ cells/well in a 96-well plate (Corning Costar, Corning, NY, USA) and allowed to adhere for 24 h. Following this incubation, the culture medium was replaced with the pre-conditioned media obtained from the material samples, and the cells were further incubated for an additional 24 h.

Cell viability was assessed using the MTT assay (Thermo Fisher Scientific, Waltham, MA, USA) according to the manufacturer ‘s instructions. Briefly, after incubation, the supernatant in each well was carefully removed and replaced with 100 μL of DMSO (Sigma Aldrich, St. Louis, MO, USA) to dissolve the formazan crystals. The optical density of the solution was measured at 570 nm using Elisa Reader (VersaMax, Molecular Devices, San Jose, CA, USA) equipment. Experiments were performed in triplicate, and the mean values of the six different readings were used for statistical analysis.

### 2.5. Statistical Procedures

Statistical analysis was performed using two-way ANOVA to evaluate the effects of the photoinitiator type and time on the degree of conversion and cell viability. Post hoc comparisons were conducted using Tukey’s HSD test, with a significance level set at *p* < 0.05.

## 3. Results

**Figure 6.**
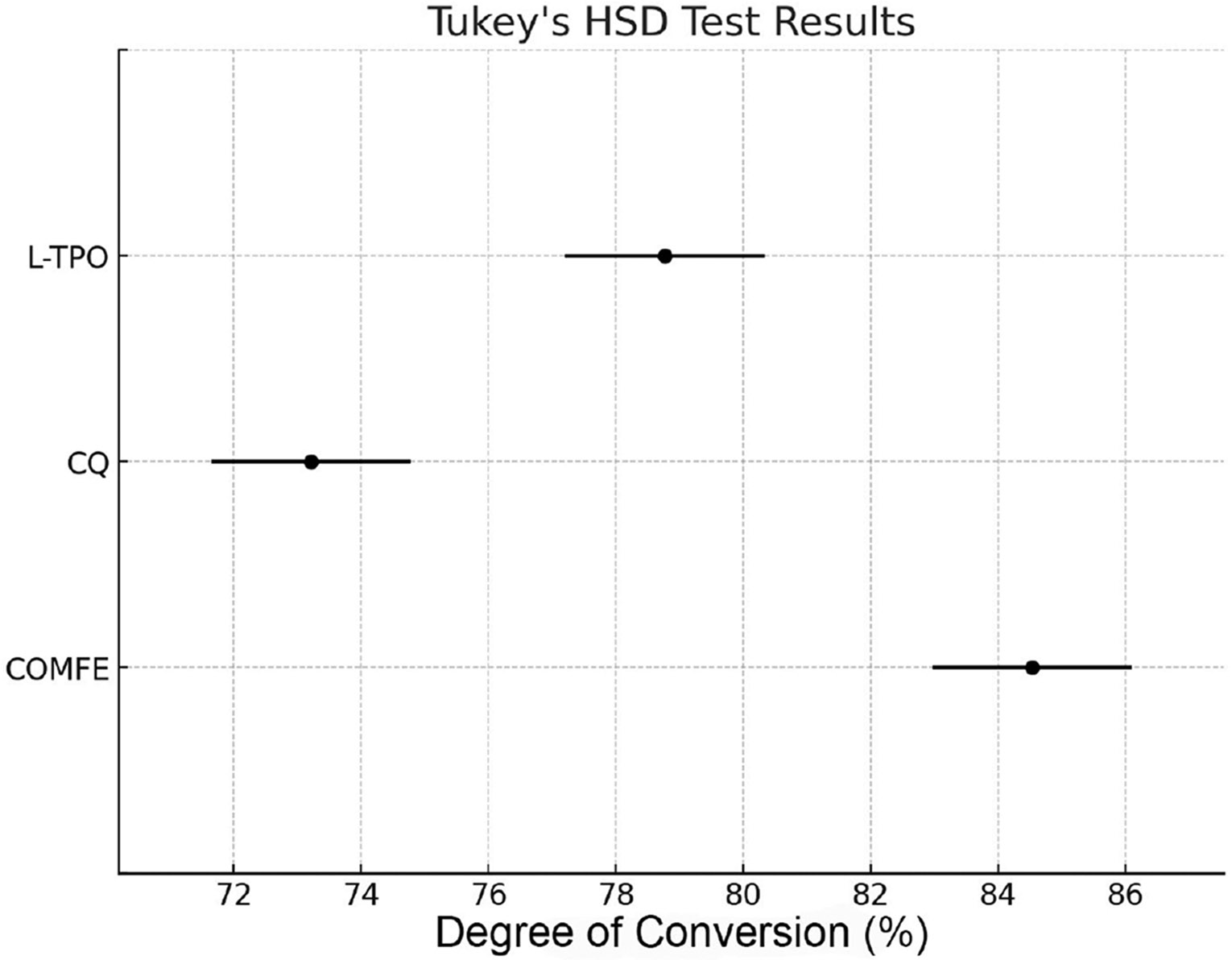
Bar chart showing the degree of conversion (%) of dental 3D-printed resin materials containing the novel Fe(II)-based coordination complex photoinitiator compared to the conventional TPO-L and CQ photoinitiators. Data are presented as mean ± standard deviation (n = 5). Statistical significance (*p* < 0.05) was found between all groups.

**Figure 7.**
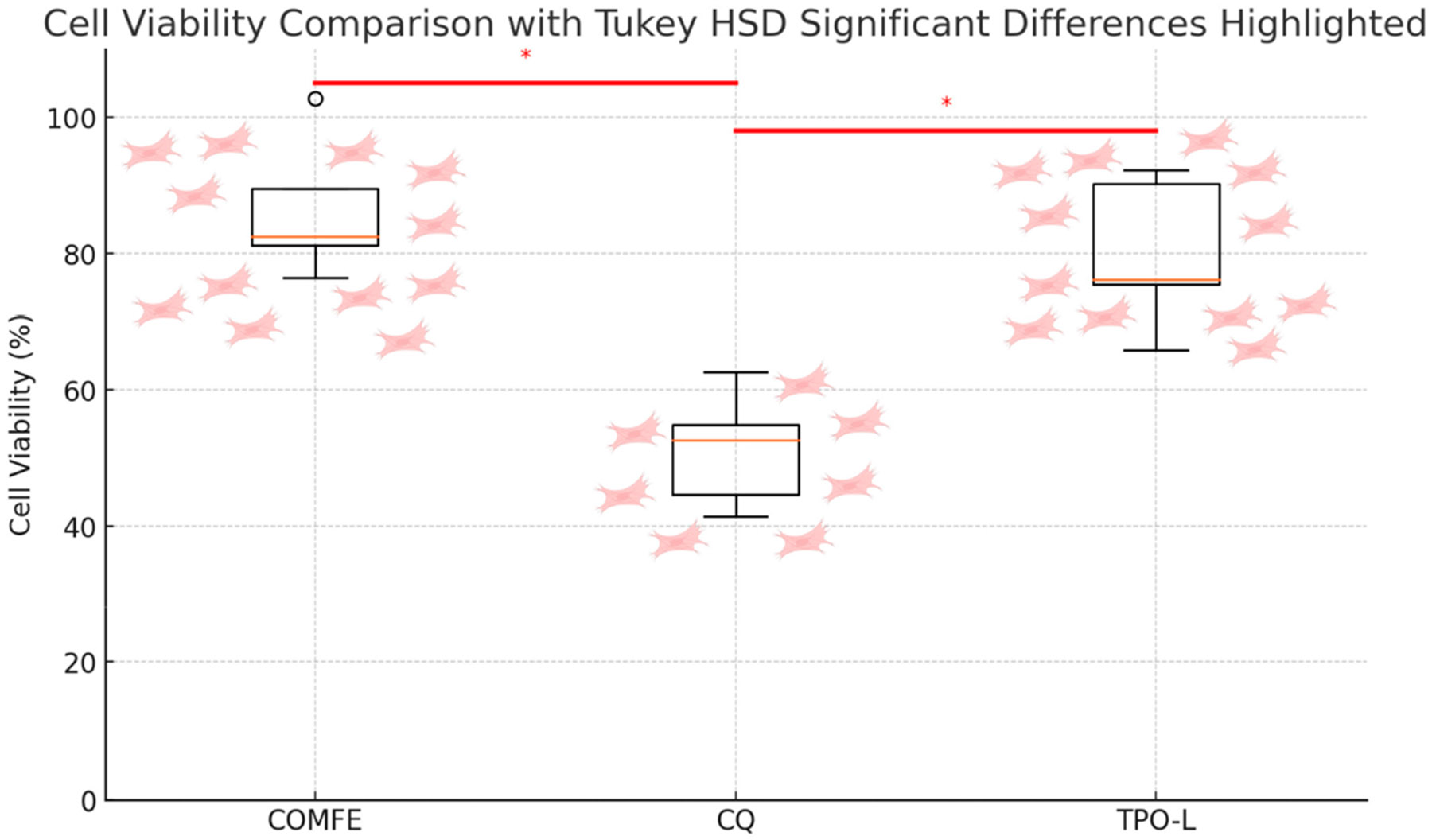
Bar chart showing the cytotoxicity (%) of L929 fibroblast-like cells when exposed to dental 3D-printed resin materials containing the novel Fe(II)-based coordination complex photoinitiator compared to the conventional TPO-L and CQ photoinitiators. Data are presented as mean ± standard deviation (n = 15). Asterisks denote statistically significant differences (* *p* < 0.05).

Figure 6 illustrates the degree of conversion (DC) for 3D-printed resins containing different photoinitiators: the novel Fe(II)-based coordination complex, TPO-L, and CQ. The results indicate that the iron-based coordination complex 3D-printed resin (mean ± SD: 84.54% ± 1.69%) showed a significantly higher DC mean value compared to the TPO-L (mean ± SD: 78.77% ± 1.25%) and CQ-based 3D-printed resins (mean ± SD: 73.21% ± 0.47%) (*p* < 0.001). The TPO-L 3D-printed resin exhibited a significantly higher DC mean value than the CQ-based 3D-printed resin (*p* < 0.001). Error bars in the figure represent the standard deviation, indicating minimal variability across replicates. Figure 7 illustrates the cell viability results from the 3D-printed resins containing the different photoinitiators. The iron-based coordination complex 3D-printed resin showed the highest cell viability at 86.5% ± 10.24%, followed by TPO-L with 80.03% ± 11.07%. CQ-based 3D-printed resin showed the lower cell viability of 51.29% ± 8.44% (*p* < 0.05), which was statistically different from the other 3D-printed resins. Error bars in the plot represent standard deviations, indicating variability within each group. The red lines and asterisks in the plot denote the statistically significant differences between these groups, which were confirmed by the Tukey HSD test. The statistical analysis confirmed a significant difference in cell viability between CQ and TPO-L and the iron-based coordination complex (*p* < 0.05). The Tukey HSD test showed that the cytotoxicity of CQ was significantly greater than the other two photoinitiators, with a Cohen’s d of 3.75 for COMFE vs. CQ and −2.92 for CQ vs. TPO-L, indicating large effect sizes. The post hoc analysis did not find a statistically significant difference between TPO-L and the iron-based coordination complex (*p* = 0.575), suggesting that both photoinitiators have comparable cytotoxic profiles.

## 4. Discussion

In this study, the cure efficiency and biocompatibility of a novel iron-based coordination complex used as a photoinitiator in comparison to the conventional TPO-L and CQ as photoinitiators in dental 3D-printed resin materials were evaluated. The first hypothesis tested was that the use of a coordination-complex-based photoinitiator would result in a higher degree of conversion compared to TPO-L and CQ. This hypothesis was accepted as we found with statistical significance that the coordination complex had the highest degree of conversion while CQ had the lowest. The degree of conversion is based upon the number of monomers that ultimately undergo polymerization reactions to form polymers [8]. This occurs when the carbon–carbon double bonds in the methylacrylate-like monomers form radicals, allowing the 3D polymerization process to form carbon–carbon single-bond polymers [1,8]. TPO-L had a higher degree of conversion than CQ because when exposed to light, TPO-L forms two active radicals, allowing for a faster polymerization reaction [8]. While CQ also generates two radicals when paired with a tertiary amine, this process is slower because it involves a reaction between two reagents after light absorption, unlike the self-cleavage mechanism of TPO-L. Moreover, only one of the radicals generated by the CQ/tertiary amine reaction is active in initiating polymerization, which further limits its efficiency, thus possibly leading to a lower degree of conversion when the two are compared [8]. On the other hand, when the iron-based coordination complex is exposed to light, it undergoes distinct photochemical reactions driven by charge transfer mechanisms. The first reaction involves homolytic bond cleavage, facilitated by metal-to-ligand charge transfer, resulting in the formation of radicals that initiate free radical polymerization. The second reaction occurs through ligand-to-metal charge transfer, where an electron and proton (hydrogen transfer) generate two distinct radicals, contributing to the polymerization process. A third possible pathway is heterolytic bond cleavage, which produces a radical species and an acid salt capable of initiating cationic polymerization [9-11]. Iron-based complexes are particularly effective due to their ability to cycle between oxidation states (e.g., Fe^2+^/Fe^3+^), which stabilizes intermediates and promotes efficient radical generation. This mechanism enables faster and more reactive polymerization compared to conventional photoinitiators like CQ and TPO-L.

CQ has an absorbance range of 360–510 nm with maximum absorbance at 470 nm. This means that it absorbs blue light. TPO-L, on the other hand, has an absorption wavelength from 350 to 430 nm with a maximum absorbance at 371 nm, meaning it absorbs UV light [8,12]. The coordination complex used in this experiment has a maximum absorbance at 570 nm, meaning that this photointitiator is absorbing at longer wavelengths. Therefore, thicker layers can be 3D printed with this photoinitiator as a longer wavelength is able to penetrate deeper through the material; conversely, CQ and TPO-L can only allow 3D printing of thinner layers due to their absorption of shorter wavelengths as shorter wavelengths have lower light penetration capabilities compared to longer wavelengths. Moreover, with a lower degree of conversion, specifically in depth, more unreacted methyacrylate-like monomers remain and can then be released to nearby surrounding tissues, which can be a concern especially for scaffold applications. These released unreacted monomers can cause local and systemic effects such as cell mutations and thereby apoptosis [7].

Thus, our second hypothesis aimed to test the biocompatibility of each photoinitiator used in this study. The second hypothesis was that the use of a coordination-complexbased photoinitiator in 3D-printed resin-based materials would result in lower cytotoxicity compared to TPO-L and CQ. This hypothesis was accepted. The results showed that CQ was the most cytotoxic photoinitiator, and that TPO-L and the iron-based coordination complex tested showed similar cytotoxicity.

The most probable cause of CQ being the most cytotoxic photoinitiator was due to the lowest degree of conversion, as demonstrated by the results from this study. A low degree of conversion results in unreacted monomers and unreacted CQ molecules, which are cytotoxic to the cells [1]. The literature shows that the photoinitiator is what is primarily released as an extract from 3D-printed resin-based materials, therefore being the main cause of cytotoxicity [1]. Even though we found no statistical differences between the iron-based-coordination complex and TPO-L, the TPO-L photoinitiator has been shown to be extremally toxic to human pulpal cells [13]. Thus, its low cytotoxicity in this study is probably explained by the high degree of conversion of the resin tested in this study. On the other hand, the iron-based coordination complex photoinitiators similar to the one tested in this study are known to have low toxicity, which is attributed to their high overall biocompatibility [9]. Upon exposure to light, coordination complexes generate superoxide radicals, which enhance their solubility and biological activity [14]. Given that photoinitiators are primarily released from 3D-printed resin-based materials, their overall biocompatibility is important depending on their application. Thus, the natural biocompatibility of coordination complexes and its widespread presence in the body can be particularly interesting for scaffold 3D printing [11,14,15]. The reason is that, in the situation of the degree of conversion being compromised, the risk of cytotoxicity is significantly lower compared to the other photoinitiators.

The results from this study provided valuable insights into 3D printing scaffolds for soft and hard tissue regeneration. Since the scaffolds act as an extracellular matrix to support the growth of new cells, they must be made of components with very low cytotoxicity or even devoid of cytotoxicity. A high degree of conversion induced by the photoinitiator reduces the amount of residual unreacted photoinitiators and monomers, which could cause cell damage and mutations [1,13]. Conversely, a low degree conversion can compromise cell growth [5,6]. Minimizing toxicity ensures that any remaining unreacted photoinitiator does not significantly harm developing cells. For those reasons, within the limitations of this study, it was possible to conclude that the iron-based coordination complex seems to be a promising photoinitiator in 3D printing as it resulted in the highest degree of conversion and similar levels of cytotoxicity as TPO-L.

One limitation of this study is that the depth of cure was not evaluated. TPO-L demonstrated a high degree of conversion, which correlated with its low cytotoxicity. However, in thicker layers, TPO-L would likely exhibit a lower degree of conversion due to its absorption of shorter wavelengths, unlike the coordination complex tested in this study. In contrast, the coordination complex, which absorbs at longer wavelengths, would maintain a high degree of conversion in thicker layers with greater depth, thus minimizing cytotoxicity. Therefore, future studies should evaluate the cytotoxicity of the coordination complexes compared to TPO-L in scenarios involving greater 3D printing layers, as this photoinitiator seems to be promising in reducing production time without compromising the depth of cure and overall cytotoxicity.

Compared to conventional photoinitiators, the novel Fe(II)-based coordination complex photoinitiator presents a safer and more efficient alternative, making it highly suitable for integration into 3D-printed scaffolds for dental applications. Future research should prioritize in vivo animal studies to confirm these findings, paving the way for clinical implementation of more biocompatible dental scaffolds.

## Data Availability

The data presented in this study are available on request from the corresponding author, as they are subject to patent rights.
